# Exercise Training Attenuates Sympathetic Activity and Improves Morphometry of Splenic Arterioles in Spontaneously Hipertensive Rats

**DOI:** 10.5935/abc.20180053

**Published:** 2018-03

**Authors:** Marina de Paiva Lemos, Gustavo Ribeiro da Mota, Moacir Marocolo, Carla Cristina de Sordi, Rosângela Soares Chriguer, Octávio Barbosa Neto

**Affiliations:** 1 Universidade Federal do Triângulo Mineiro (UFTM), Uberaba, MG - Brazil; 2 Universidade Federal de Juiz de Fora (UFJF), Juiz de Fora, MG - Brazil; 3 Universidade Federal de São Paulo, São Paulo, SP - Brazil

**Keywords:** Exercise, Physical Exertion, Hypertension, Vascular Resistance, Arterioles, Rats

## Abstract

**Background:**

Alterations in the structure of resistance vessels contribute to elevated systemic vascular resistance in hypertension and are linked to sympathetic hyperactivity and related lesions in target organs.

**Objective:**

To assess the effects of exercise training on hemodynamic and autonomic parameters, as well as splenic arteriolar damages in male Wistar Kyoto (WKY) and Spontaneously Hypertensive Rats (SHR).

**Methods:**

Normotensive sedentary (WKY_S_) and trained (WKY_T_) rats, and hypertensive sedentary (SHR_S_) and trained (SHR_T_) rats were included in this study. After 9 weeks of experimental protocol (swimming training or sedentary control), arterial pressure (AP) and heart rate (HR) were recorded in freely moving rats. We assessed the autonomic control of the heart by sympathetic and vagal autonomic blockade. Morphometric analyses of arterioles were performed in spleen tissues. The statistical significance level was set at p < 0.05.

**Results:**

Resting bradycardia was observed in both trained groups (WKY_T_: 328.0 ± 7.3 bpm; SHR_T_: 337.0 ± 5.2 bpm) compared with their respective sedentary groups (WKY_S_: 353.2 ± 8.5 bpm; SHR_S_: 412.1 ± 10.4 bpm; p < 0.001). Exercise training attenuated mean AP only in SHR_T_ (125.9 ± 6.2 mmHg) vs. SHR_S_ (182.5 ± 4.2 mmHg, p < 0.001). The WKY_T_ showed a higher vagal effect (∆HR: 79.0 ± 2.3 bpm) compared with WKY_S_ (∆HR: 67.4 ± 1.7 bpm; p < 0.05). Chronic exercise decreased sympathetic effects on SHR_T_ (∆HR: -62.8 ± 2.8 bpm) in comparison with SHR_S_ (∆HR: -99.8 ± 9.2 bpm; p = 0.005). The wall thickness of splenic arterioles in SHR was reduced by training (332.1 ± 16.0 µm^2^ in SHR_T_ vs. 502.7 ± 36.3 µm^2^ in SHR_S_; p < 0.05).

**Conclusions:**

Exercise training attenuates sympathetic activity and AP in SHR, which may be contributing to the morphological improvement of the splenic arterioles.

## Introduction

Essential hypertension is inwardly connected to the blood vessels and is characterized by chronic increases in peripheral vascular resistance, mainly resulting from functional and structural alterations of the microcirculation. These lesions can be both the cause and the consequence of the elevation of arterial pressure (AP).^1^ The major pathways that interact to develop morphological changes in arteriolar vessels in hypertension may compromise the splenic vessels (arteriolar hyalinosis, fibrinoid necrosis) and the interstitial space, causing fibrosis.^2-5^ The arteriolar hyalinosis occurs by filtration of plasma proteins through the endothelium. It is not exclusive of any disease, being observed in arterioles of normal aging, especially in arterioles of the spleen. However, it occurs earlier and more intense in arterial hypertension.^6^

The autonomic nervous system plays a key role in the stabilization of AP control for maintaining homeostasis. In this respect, the literature data show that the sympathetic nervous system (SNS) can reciprocate incisively in the development of some forms of hypertension. Evidence of the participation of this system in the control of normal cardiovascular and metabolic functions and its role in the genesis and maintenance of several diseases is broad. The importance of understanding the workings of the SNS and systems related to it is essential not only to elucidate the path physiology of some diseases, but to understand how drugs that act on the adrenergic system interfere with the evolution of pathologies significantly altering the prognosis of patients.^7^

Experimental evidence has shown that chronic exercise produces beneficial effects on the cardiovascular system via alterations in neural control of the circulation. These effects include reductions in AP, sympathetic activity^8^ and vascular resistance^9^ concomitantly with attenuation in the target-organ damage.^10^ If there is relation between exercise training and decrease of vascular resistance, the mechanisms by which chronic exercise training improves splenic arteriolar morphometry are not well established. Thus, the aim of this study was to assess the effects of exercise training on sympathetic activity and arteriolar damages in spleens of spontaneously hypertensive rats (SHR).

## Methods

### Animal model and exercise training protocol

Forty male SHR and Wistar Kyoto rats (WKY) aged 45-50 weeks were randomly assigned into four experimental groups of 10 rats each: SHR_T_ and WKY_T_ (that were submitted to exercise training protocol by swimming) or SHR_S_ and WKY_S_ (that were kept sedentary for a similar period of time). The sample size (n) was determined based on studies that evaluated the effects of exercise training on hypertension. These studies served as the basis for the present study that investigates the cardiovascular effects of the accumulated exercise.^11,12^ All animals were kept in grouped cages (n = 3) at room temperature around 23ºC, humidity of 40-70% and photoperiod of 12-hour light/dark cycle. Efforts were made to avoid any unnecessary distress to the rats, in accordance to the Brazilian Council for Animal Experimentation. All animal protocols were approved by the local Experimental Animal Use Committee (#271/2013), and were performed according to the regulations set forth by the National Institutes of Health Guidelines for the Care and Use of Laboratory Animals.

The swimming exercise protocol was performed in a glass tank and ambient water temperature was kept at 30º ± 1ºC. The trained animals received a 20-min adaptation period on the first day, with increases of 10 min each day until reaching 1 hour on the fifth day.^13^ After this period, the rats trained 5 days/week with a gradual progression toward a 2-hour session during nine weeks. This protocol is defined as an aerobic endurance and low-intensity training, as the animals swam without additional work load, this method corresponds the intensity below the anaerobic threshold in rats.^14^ Sedentary animals were placed in the swimming apparatus for 10 min twice a week to mimic the water stress associated with the experimental protocol.

### Surgical procedures and hemodynamic parameters recording

Twenty-four hours after the last exercise training session, all animals were anesthetized with sodium pentobarbital (40 mg/kg ip) and cannulas of polyethylene (PE-10) were implanted into the femoral artery for cardiovascular recording and into the femoral vein for drug infusion. Then, the polyethylene catheters were exteriorized at the posterior neck region of the animal. Rats received food and water *ad libitum* and were studied 1 day after catheter placement. Prophylactic treatment with antibiotics and anti-inflammatory drugs were performed to prevent postsurgical infections and inflammation, respectively.^15^ After 48 hours of recovery from the anesthesia and surgery, the arterial cannula was connected to an AP transducer and a signal amplifier (Model 8805A, Hewlett-Packard, USA) was converted by the analog-digital signal plate (sampling frequency - 1000 Hz) by a computerized system data acquisition (Aqdados, Tec Lynx. Eletron. SA, Sao Paulo, Brazil) and stored on computer. The animals were maintained in a peaceful environment for a period of 15 minutes and adaptive later pulsatile AP was continuously recorded at baseline for 30 minutes. During the experimental procedure, systolic AP (SAP), diastolic AP (DAP), mean AP (MAP) and heart rate (HR) were derived from pulsatile AP.

### Cardiac autonomic tonus

To evaluate the exercise training influence on the tonic autonomic control of the heart, we also performed the sympathetic and vagal autonomic blockade after propranolol (5 mg/kg, i.v.) and atropine (4mg/kg, i.v.) injections, respectively, to calculate the sympathetic and vagal effects, as well as the intrinsic HR (iHR) and tonic sympathovagal index.^14^ The autonomic blockers were administered in a random sequence with a 15-min interval between them. After double blockade, the cardiovascular recordings lasted for 15 min. Briefly, the sympathetic effect was considered as the difference between the HR after sympathetic blockade and resting HR. Vagal effect was calculated as the difference between HR after vagal blockade and resting HR. The tonic sympathovagal index was obtained as the ratio between resting HR and iHR, considering that the iHR was the HR obtained after double autonomic blockade.^16^

### Analysis of splenic arteriolar morphometry

All animals were anesthetized with sodium pentobarbital and euthanatized with a lethal dose of potassium chloride. Their spleens were excised postmortem and immersed in saline (0.9%) to remove excess blood. Shortly after, the organs were placed on foil, previously treated and weighed in a semi-analytical Gehaka BG2000^®^. Subsequently, the material was cut and placed inside a sterilized glass with 10% formaldehyde. Thereupon, the material was dehydrated using ethanol at concentrations of 80%, 90% and 95%. Diaphanization was performed with xylol. The material was placed in containers containing liquid paraffin at 60ºC. Then, the material was placed in blocks. Histological 2-µm cuts were performed using a microtome and then the material were mounted in glass slides and stained with Masson's Trichrome Blue. The area of the inner and outer layers of each arteriole was quantified by using common light microscope for capturing the images and the imageJ program to check the area of each layer. At the end of the procedures for quantification of the area of each layer, the thickness of each arteriole was obtained.

### Statistical analysis

Shapiro-Wilks and Levene's tests were used to evaluate the normality and homogeneity of the sample. Results were expressed as mean ± SD (for normally distributed variables) or median with upper and lower quartiles (for non-normally distributed variables). For parametric data, we used two-way ANOVA (etiology vs. intervention), with the Tukey as a post hoc test. The nonparametric data were analyzed by the Mann-Whitney test. Pearson coefficient was used to test the correlation between sympathetic effect with area of outer wall thickness and total area thickness. Probability values of P < 0.05 were considered statistically significant. Analyses were performed using SigmaStat® v. 2.03 (SPSS, Chicago, IL, USA).

## Results

**Figure 1:**
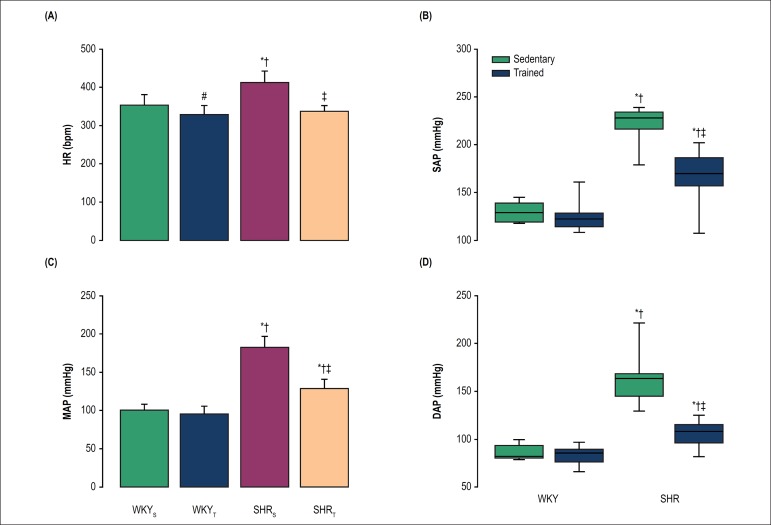
Baseline recording of heart rate (1A), systolic arterial pressure (1B), mean arterial pressure (1C) and diastolic arterial pressure (1D) in freely moving rats. WKY_S_ (sedentary normotensive rats); WKY_T_ (trained normotensive rats); SHR_S_ (sedentary hypertensive rats); SHR_T_ (trained hypertensive rats). Bars in figures 1A and 1C represent mean ± SD. Results in figures 1B and 1D are expressed as median (interquartile range). ^#^p < 0.05 vs. WKY_S;_ *p < 0.001 vs. WKY_S_; ^†^p < 0.001 vs. WKY_T_ and ^‡^p < 0.001 vs. SHR_S_.

The SHR_S_ showed higher resting HR in comparison to WKY_S_ (p < 0.001). As expected, both trained groups presented higher resting bradycardia compared with their respective sedentary groups (p < 0.001; Figure 1A).

Exercise training also was able to decrease baseline SAP (p < 0.001; Figure 1B), MAP (p < 0.001; Figure 1C) and DAP (p < 0.001; Figure 1D) in hypertensive animals compared with their respective sedentary group. The SHR_S_ presented higher pressure levels than WKY_S_ (p < 0.001) and WKY_T_ (p < 0.001) groups. After the 9-week training period, the AP was similar in WKY_T_ and WKY_S_.

**Figure 2:**
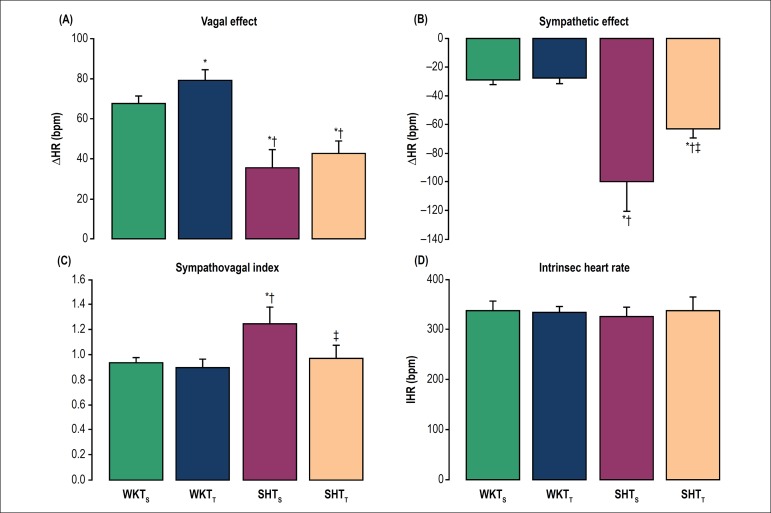
Effects of exercise training on the tonic autonomic control of the heart rate (HR) in non-anesthetized rats. (2A) vagal and (2B) sympathetic effects were obtained, respectively, by the difference between vagal blockade (by atropine) or sympathetic blockade (by propranolol) and resting HR. (2C) Sympathovagal balance was expressed by the tonic sympathovagal index, which is the ratio between resting and intrinsic HR (iHR). (2D) Intrinsic HR (bpm) obtained after autonomic double pharmacological blockade. Bars represent mean ± SD. *p < 0.05 vs. WKY_S;_^†^p < 0.05 vs. WKY_T_ and ^‡^p < 0.05 vs. SHR_S_.

To evaluate the influence of chronic exercise on the tonic autonomic control of the heart, we performed the vagal and sympathetic autonomic blockade with atropine and propranolol injections, respectively, to calculate the vagal (Figure 2A) and sympathetic effects (Figure 2B), as well as the tonic sympathovagal index (Figure 2C) and iHR (Figure 2D). No difference on vagal effect was observed between the hypertensive groups. However, the WKY_T_ group evidenced a higher vagal effect than the WKY_S_ group (p < 0.05). Both hypertensive groups presented a lower vagal effect when compared with their respective normotensive groups (p < 0.001). In addition, no difference in the sympathetic effect was observed between the normotensive groups (p = 0.563). On the other hand, the SHR_T_ group showed a lower sympathetic effect as compared with SHR_S_ group (p = 0.005). Both normotensive groups had a lower sympathetic effect when compared with their respective hypertensive groups (p < 0.001). The sympathovagal index was lower in SHR_T_ than in SHR_S_ (p < 0.05). No difference was observed between the groups regarding iHR.

Morphometric analysis after histological processing revealed profound changes in microcirculatory profile of spleen circulation induced by training in hypertensive animals (Table 1). As expected, hypertensive splenic arterioles had a thicker wall than normotensive arterioles (p < 0.001). Despite this, exercise training was effective to normalize SHR arteriole wall/lumen ratio in spleen tissues analyzed when compared with that of SHR_S_ (p < 0.001). The SHR_S_ also presented a greater area of outer wall thickness when compared to WKY_S_ and WKY_T_ (p < 0.001). After exercise training protocol, the SHR_T_ obtained a reduction in the area of the outer wall thickness compared to SHR_S_ (p < 0.001). Similar results were observed in the total area thickness. The SHR_S_ had a higher total area thickness of the splenic arterioles than the normotensive groups (p < 0.005). In addition, the SHR_T_ evidenced an attenuation in total area thickness of splenic arterioles when compared with SHR_S_ (p < 0.005).

**Table 1:** Values related to morphological analysis of the area of the wall thickness of splenic arterioles.

Thickness area	WKY_S_ (n = 10)	WKY_T_ (n = 10)	SHR_S_ (n = 10)	SHR_T_ (n = 10)
Inner wall (µm^2^)	60.5 ± 3.4	58.8 ± 2.3	87.3 ± 3.3*^†^	58.0 ± 2.6^‡^
Outer wall (µm^2^)	419.8 ± 29.3	405.6 ± 21.7	632.4 ± 29.1*^†^	418.8 ± 16.4^‡^
Total area (µm^2^)	335.6 ± 44.7	349.7 ± 35.8	502.7 ± 36.3*^†^	332.1 ± 16.0^‡^

Data are expressed as mean ± SD. Abbreviations: WKY_S_, sedentary normotensive rats; WKY_T_, trained normotensive rats; SHR_S_, sedentary hypertensive rats; SHR_T_, trained hypertensive rats. Data expressed as mean ± SEM

* p < 0.05 vs. WKY_S_;

† p < 0.05 vs. WKY_T_ and

‡ p < 0.05 vs. SHR_S_.

**Figure 3:**
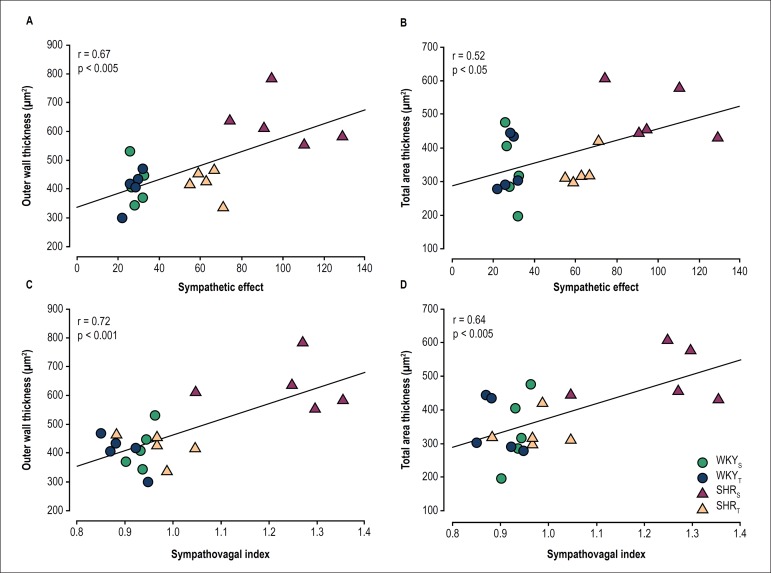
Correlation coefficient between sympathetic effect and outer wall thickness (A), sympathetic effect and total area thickness (B), sympathovagal index and outer wall thickness (C), sympathovagal index and total area thickness (D).

Further analysis showed a significant association between sympathetic effect and area of outer wall thickness (r = 0.67, p < 0.005; Figure 3A), sympathetic effect and total area thickness (r = 0.52, p < 0.05; Figure 3B), sympathovagal index and area of outer wall thickness (r = 0.72, p < 0.001; Figure 3C) and sympathovagal index and total area thickness (r = 0.64, p < 0.005; Figure 3D).

## Discussion

Our main findings confirmed the efficacy of exercise training to attenuate sympathetic overactivity and to lower AP in hypertensive animals, showing, in addition, that the training-induced, sympathetic-lowering effect was associated with normalization of abnormal splenic artery diameter, decreasing the degree of vascular injury in spleen. The morphometric analysis of small vessels employed in the present study revealed that the splenic vascular adjustments are specific for the SHR_T_. It is well documented that chronic physical exercise attenuates sympathetic hyperactivity^10^ and arteriolar damage on hypertension.^17^ To our knowledge, however, this is one of the first reports to evidence association between a reduction in splenic arteriole injury and sympathetic activity.

The cause-effect relation between hypertension and arteriolar damage (hypertrophy) is well established.^18-20^ In this sense, the literature evidences that an effective antihypertensive treatment should aim not only to reduce AP but also to correct injuries associated with hypertension, such as the altered vascular structure. A previous study has shown the efficacy of training to normalize arteriole wall/lumen ratio, evidencing that arteriolar response as well as vascular resistance reduction after exercise training were significantly correlated with AP reduction.^21^ Experimental study has found that arteriole wall/lumen ratios were reduced by increased internal and/or external diameter, which is a characteristic pattern for vascular remodeling.^21^ Of importance is the demonstration that exercise training, by reversing lumen encroachment, normalizes enlarged wall/lumen ratio of small arterioles in hypertensive rats. These data are in accordance with the results found in our study.

Results from studies with animal models indicate that a sustained elevation of sympathetic tonus stimulates smooth muscle cell hypertrophy, suggesting that sympathetic overactivity may contribute to changes in arterial wall thickness.^22^ In this way, an interesting finding in our study was a positive and significant correlation between sympathetic hyperactivity and splenic arterioles wall thickness in hypertensive rats, corroborating with results from other investigators who demonstrated that hypertension is associated with sympathetic overactivity that alters vasomotor control resulting in several abnormalities in tissue microcirculation, such as increased arteriolar wall-to-lumen ratio and decreased vessel density, which contribute to maintain an elevated total peripheral resistance.^23-28^ Another important finding in our research was that exercise training was able to attenuate sympathetic activity in SHR and that this effect was associated with a reduction in splenic arteriole wall thickness. Exercise training produces beneficial effects on cardiovascular system in normal and sick people via alterations (or modifications) in the neural control of circulation.^29,30^ These effects include reductions in AP, sympathetic outflow in humans,^31,32^ as well as in animal models,^33,34^ and vascular resistance.^35,36^ In addition, there is evidence that exercise training improves the conditions of the small vessels in SHR subjected to swimming protocol.^37^ Although this study did not address the mechanisms responsible for training-induced effects, one might speculate that arteriole adjustments are group-specific (hypertensive rats) and probably not dependent on paracrine, autocrine, metabolic, and/or myogenic factors, since similar alterations were observed in a previous study.^17^

It is well established that regular physical activity reduces AP in hypertensive individuals, without significant pressure changes in normotensive individuals.^38-40^ In fact, several studies have suggested that exercise training intensity influences the pressure-lowering effect, with larger reductions being observed with lower exercise intensities.^40^ We did not analyze the effect of training intensity, but our results clearly showed that the exercise protocol used caused an important AP decrease only in the SHR group. Pressure reduction was accompanied by both resting bradycardia and specific training-induced adjustment in splenic hypertensive arterioles. Resting bradycardia is considered to be an excellent hallmark for exercise training adaptation in humans and rats.^39-40^ Thus, the bradycardia found in trained rats clearly demonstrates the effectiveness of the exercise protocol here used.

## Conclusion

Considering our findings, we can conclude that exercise training was effective in reducing AP and improving splenic arteriolar morphometry in hypertensive rats. Briefly, these data strongly suggest that this improvement was associated with decreased sympathetic nerve activity. In addition, regression of hypertrophied splenic arteriole is the anatomic response to exercise training specific to the SHR group. These compensatory adjustments, by reducing local resistance and augmenting physical capacity, contribute to the training-induced, pressure-lowering effect observed in hypertensive individuals.
